# Supplementary material for: Gut commensal Alistipes shahii improves experimental colitis in mice with reduced intestinal epithelial damage and cytokine secretion
Source: mSystems. 2025 Feb 12;10(3):e01607-24. doi: 10.1128/msystems.01607-24 (PMC11915872; doi:10.1128/msystems.01607-24)
Supplement: Supplemental material — Legends for Figures S1 and S2; Tables S1 and S2. [file msystems.01607-24-s0003.docx]

**Supplemental material**

Legends for Figure S1-S2; Tables S1-S2.

**Figure S1** Weight change for 7 days in the pre-gavage stage.

**Figure S2** Rarefaction curves.

**Table S1** Antibiotic susceptibilities of *A. shahii* isolates by E-test

| Antibiotics | As274 | As355 | As360 | As364 | QC^#^ |
| --- | --- | --- | --- | --- | --- |
| Amoxicillin/clavulanate | R^$^ | R | S | S | S |
| Clindamycin | R | R | S | R | S |
| Imipenem | S | S | S | S | S |
| Meropenem | S | S | S | S | S |
| Metronidazole | S | S | S | S | S |
| Penicillin | S | R | S | S | R |
| Ampicillin | S | R | S | S | R |
| Ceftriaxone | S | R | S | S | R |
| Chloramphenicol | R | R | S | S | S |
| Moxifloxacin | R | R | S | R | S |

^#^QC, quality control strain: *Bacteroides fragilis* ATCC 25285.

^$^R, resistant; S, susceptible.

**Table S2** SCFAs content and pH value in the culture supernatants of *A. shahii* As360.

| strain | pH | | SCFAs content (μg/mL) | | | | | | |
| --- | --- | --- | --- | --- | --- | --- | --- | --- | --- |
|  | 0 h | 48 h | Acetic acid | Propionic acid | Isobutyric acid | Butyric acid | Isovaleric acid | Valeric acid | Hexanoic acid |
| As360 | 7.54±0.05 | 5.71±0.01 | 481.48 | 599.67 | 12.98 | 2.97 | 48.46 | 0.08 | 0.59 |
